# A unified understanding of the human mind — a neuroethical perspective: Tracing the evolution in western thought and the integration with neuroscience, psychology, psychiatry, and relational dimensions

**DOI:** 10.1017/S109285292400049X

**Published:** 2024-12-02

**Authors:** Alberto Carrara

**Affiliations:** 1 Faculty of Philosophy and Neurobioethics Research Group (GdN), Pontifical Athenaeum Regina Apostolorum (APRA), Rome, Italy; 2 UNESCO Chair in Bioethics and Human Rights, Rome, Italy; 3 Faculty of Psychology, European University of Rome (UER), Rome, Italy

**Keywords:** philosophy, neuroscience, neuroethics, neurobioethics, psychiatry, human person

## Abstract

This article, titled “A Unified Understanding of the Human Mind - A Neuroethical Perspective,” examines the evolution of the concept of the human mind in Western thought and its integration with neuroscience, psychology, psychiatry, and relational dimensions. The author explores how the understanding of the mind has changed over time, influenced by shifts in philosophical paradigms, scientific advancements, and societal perspectives. The article traces the historical development of the mind’s concept, starting from ancient Greece, through influential thinkers like Plato and René Descartes, and progressing to contemporary perspectives. It highlights various philosophical and scientific approaches, including structuralism, functionalism, empiricism, and associationism, which have shaped our understanding of the mind. The article also delves into contemporary integration, where advancements in neuroimaging and the rise of holistic approaches offer a more nuanced understanding of the human mind. The author emphasizes the importance of the relational dimension and the interconnectedness of mental processes, the brain, and the external environment. This integrated perspective can benefit psychiatric treatment and psychological assessments by fostering a holistic approach to mental health. In conclusion, the article advocates for a multidimensional perspective that bridges subjective and objective aspects of human experience, offering promise for theoretical knowledge and practical applications in psychology, psychiatry, and neuroscience.

## Introduction

The concept of the mind has been a central focus of intellectual inquiry in the Western world, with its evolution marked by a rich tapestry of philosophical, psychological, and scientific developments. From ancient Greece to the present day, the understanding of the mind has undergone significant transformations, reflecting shifts in philosophical paradigms, scientific advancements, and societal perspectives.

This article explores the historical trajectory of the concept of human mind in the Western context, leading to a contemporary synthesis that integrates neuroscientific data, psychological and psychiatric orientations, and the relational dimension. This emerging framework on the reality of the human mind represents a valuable tool to validate a specific psychiatric treatment or a concrete psychological assessment.

## The human mind in history

1. Ancient Greece: In ancient Greece, the exploration of the mind (ψυχή) marked a foundational chapter in Western philosophical thought. Early thinkers like Thales, Anaximenes, and Heraclitus sought to unravel the mysteries of human consciousness, offering diverse theories that spanned the spectrum from materialistic to metaphysical–spiritual explanations. Thales, for instance, speculated about water as the fundamental substance underlying all existence, including mental phenomena. Anaximenes, in turn, proposed air as the essential element shaping the mind.
  However, the seminal contributions of these early philosophers paved the way for a more intricate understanding of the mind through the works of Plato. The body–mind problem is one of the most fundamental philosophical questions, which concerns the nature and relationship of the human mind and body. Plato’s dialogue *Alcibiades I* (On human’s nature) explores this problem through the conversation between Socrates and the young and ambitious Alcibiades, who wants to become a great leader of Athens. Socrates challenges Alcibiades to examine himself and his own nature before he can rule others or engage in politics. He argues that self-knowledge is the most important and divine kind of knowledge, and that it requires looking at the soul, which is the true self of a person.^1^ The dialog presents three possible solutions to the body–mind problem, which can be called the dualistic, the monistic, and the unidual solutions. The dualistic solution is based on the idea that the mind and the body are two distinct and separate entities (or substances or *res*, in Latin), and that the mind is superior to the body in every way. The mind is immortal, divine, rational, and beautiful, while the body is mortal, material, irrational, and ugly. The mind is the source of virtue and wisdom, while the body is the source of vice and ignorance. The mind is the true self, it is the human being, while the body is a mere instrument or prison for the mind.^2^ The dualistic solution implies that the mind should detach itself from the body as much as possible and seek to contemplate the eternal and intelligible forms, which are the objects of true knowledge. The monistic solution is based on the idea that the mind is not real, but it is just an epiphenomenon of the material constitution of the body. This type of monistic interpretation is called materialistic monism, and it is very present in nowadays society. Finally, the unidual solution of the mind–body problem is based on the idea that the mind and the body are not separate, but rather aspects of the same entity, which is the human being. The mind and the body are both natural and necessary parts of the human nature, and they both contribute to the human excellence and happiness. The mind is not superior to the body, but rather depends on the body for its proper functioning and development.
  Plato’s philosophical legacy is notably encapsulated in his theory of the tripartite soul, expounded in works such as *Phaedrus* and *The Republic.* Departing from the monistic inclinations of his predecessors, Plato introduced a dualistic perspective that delineated the mind into three distinct components: the rational, the spirited, and the appetitive. Plato’s tripartite soul concept was a metaphorical construct designed to illuminate the complex dynamics of human psychology. The rational component, located in the head, represented the intellect and reason, governing logical thought processes. The spirited aspect, residing in the chest, embodied the emotional and courageous dimensions of the psyche, steering the individual toward honor and virtue. Lastly, the appetitive element, situated in the abdomen, symbolized primal desires and appetites, encompassing basic needs and passions.
  Plato’s dualistic framework, while rooted in abstract metaphors, laid the groundwork for later philosophical discussions on the mind–body relationship. The tripartite soul not only influenced subsequent Hellenistic and Roman philosophies but also resonated across centuries, leaving an enduring impact on Western thought. This dualistic perspective, emphasizing the interplay of reason, emotion, and desire, set the stage for future inquiries into the nature of the mind, ultimately contributing to the rich tapestry of ideas that shaped the evolution of Western philosophical and psychological thought.
2. Cartesian Dualism: René Descartes, in the 17th century, furthered the dualistic tradition by asserting a strict separation between mind and body. Cartesian dualism posited that the mind (*res cogitans*) and the body (*res extensa*) were distinct entities, with the mind serving as the seat of consciousness and reason. This separation influenced Western thought for centuries. In the 17th century, René Descartes made a profound impact on the understanding of the mind through his formulation of Cartesian dualism, a philosophical stance that fortified and extended the dualistic tradition in Western thought. Descartes, often regarded as the father of modern philosophy, departed from the prevailing ideas of his time by asserting a rigorous and unequivocal separation between the mind and the body. Descartes’ dualistic framework is encapsulated in his famous assertion, *Cogito, ergo sum* (I think, therefore I am). According to Cartesian dualism, the mind, referred to as *res cogitans* or the thinking substance, and the body, termed *res extensa* or the extended substance, were fundamentally distinct entities. The mind, in this paradigm, was conceptualized as the seat of consciousness, self-awareness, and reason, while the body was considered a mechanical, extended entity governed by physical laws. Descartes’ emphasis on the separation of mind and body was motivated by a desire to establish a foundation for certain knowledge. By isolating the mind as a thinking substance, he sought to ground human certainty in the realm of reason, thereby initiating a methodical and systematic approach to understanding reality. This conceptual division also allowed Descartes to reconcile the incorporeal nature of the mind with the materialistic aspects of the body, providing a framework that aligned with the scientific inquiries of his era. The influence of Cartesian dualism extended far beyond Descartes’ immediate intellectual milieu, permeating Western thought for centuries. This dualistic perspective profoundly impacted the fields of philosophy, science, and psychology, setting the stage for ongoing debates regarding the mind–body relationship. While Descartes’ approach brought clarity to philosophical discourse, it also fostered a dichotomous view that hindered a more holistic understanding of human nature. Critics of Cartesian dualism argue that such a strict separation oversimplifies the complexities of the mind–body interaction, neglecting the intricate interconnections between mental and physical phenomena. Nevertheless, Descartes’ legacy remains palpable in the enduring dichotomy between mind and body that continues to shape contemporary philosophical and scientific discussions. The Cartesian dualistic tradition, with its enduring impact, serves as a critical juncture in the historical trajectory of Western conceptions of the mind.
3. Empiricism and Associationism: The empiricist tradition, championed by philosophers like John Locke and David Hume, shifted the focus from innate ideas to the mind’s dependence on sensory experience. The associationist perspective, as articulated by thinkers like David Hartley and James Mill, emphasized the role of associations in mental processes, laying the groundwork for a more empirical understanding of the mind. The empiricist tradition, a pivotal chapter in the history of Western thought on the mind, emerged as a reaction against the rationalist doctrines that posited innate ideas and a priori knowledge. Spearheaded by luminaries such as John Locke and David Hume, empiricism redirected attention toward the notion that the mind is a tabula rasa, a blank slate shaped by sensory experiences and external stimuli. John Locke, often regarded as the founder of empiricism, contended that the mind at birth is void of any innate ideas and that all knowledge is derived from sensory impressions. In his *Essay Concerning Human Understanding*, Locke proposed that the mind is like an empty vessel that gradually accumulates knowledge through sensory experiences, thereby emphasizing the importance of observation, perception, and reflection in the formation of ideas. For Locke, the human person is the reasoning and self-conscious entity which is independent of the body.^1^^,^^3^
  David Hume, a prominent figure in the Scottish Enlightenment, took Locke’s empiricism to new heights by challenging the concept of causation and questioning the nature of reality itself. In his *A Treatise of Human Nature*, Hume delved into the idea that all knowledge is based on impressions and ideas, rejecting the existence of causally connected entities. Hume’s radical empiricism undermined traditional notions of causation and fueled skepticism, reshaping the landscape of epistemology and the philosophy of mind. The associationist perspective, intricately linked with empiricism, found expression through the works of thinkers like David Hartley and James Mill. David Hartley, in his *Observations on Man, His Frame, His Duty, and His Expectations*, proposed a psychological theory based on the principle of associationism. Hartley argued that mental phenomena, including thoughts and emotions, result from the association of sensory experiences, forming complex chains of ideas. James Mill, a utilitarian philosopher and father of John Stuart Mill, further developed the associationist framework by positing that mental processes are composed of elementary ideas linked through associative principles. Mill’s contributions laid the groundwork for a more systematic and empirical understanding of the mind, wherein complex mental phenomena could be dissected into simpler components governed by principles of association. The empiricist and associationist tradition, championed by Locke, Hume, Hartley, and Mill, shifted the intellectual landscape away from innate ideas toward an empirical investigation of the mind’s reliance on sensory experiences and the associative processes governing mental life. This paradigmatic shift not only influenced the trajectory of philosophy but also paved the way for the empirical methods embraced by modern psychology, marking a significant transition in the evolution of Western thought on the nature of the mind.
4. Structuralism and Functionalism: In the late 19th century, psychology emerged as a distinct scientific discipline. Structuralists like Wilhelm Wundt sought to analyze the mind’s structure through introspection, while functionalists like William James focused on understanding the mind’s adaptive functions. This period marked the beginnings of a more systematic and empirical approach to the study of the mind. The late 19th century witnessed the birth of psychology as a distinct scientific discipline, marking a departure from philosophical speculation to a more systematic and empirical inquiry into the intricacies of the mind. This transformative period gave rise to two influential schools of thought: structuralism, led by Wilhelm Wundt, and functionalism, championed by William James. Wilhelm Wundt, often regarded as the father of experimental psychology, founded the first psychological laboratory in 1879 at the University of Leipzig. Wundt’s structuralist approach aimed to unravel the complexities of the mind by employing a method known as introspection. Subjects were instructed to reflect on and report their own thoughts, feelings, and sensations in response to controlled stimuli. Through these introspective analyses, Wundt sought to identify the fundamental elements, or structures, of consciousness. His emphasis on rigorous observation and experimental procedures laid the foundation for psychology as a scientific discipline. In contrast to Wundt’s structuralism, William James, a pioneering American psychologist, spearheaded the functionalist movement. James shifted the focus from the mere analysis of the mind’s structure to understanding the adaptive functions of mental processes. His seminal work, *The Principles of Psychology*, explored how the mind functions to help individuals adapt to their environment. James was particularly interested in the evolutionary advantages conferred by various mental phenomena, emphasizing the practical utility of consciousness and behavior. Functionalism broadened the scope of psychological inquiry, incorporating the study of emotions, habits, and practical problem-solving into the discipline. The structuralist and functionalist perspectives, though distinct, shared a commitment to empirical investigation and the scientific study of the mind. Structuralism sought to identify the elemental building blocks of consciousness, paving the way for systematic analysis. Meanwhile, functionalism focused on the adaptive roles of mental processes, aligning psychology with evolutionary principles and emphasizing the pragmatic significance of cognitive functions. This period marked a pivotal shift in the history of psychology, transitioning from speculative and introspective approaches to a more rigorous, empirical, and experimental discipline. The legacy of structuralism and functionalism endured, influencing subsequent psychological schools and shaping the development of methodologies that remain integral to contemporary psychological research. The dialectical interplay between these two schools laid the groundwork for the multifaceted and interdisciplinary nature of modern psychology, reflecting the evolving quest to comprehend the intricate workings of the human mind.
5. Expressive individualism: according to O. Carter Snead, a legal scholar and bioethicist, Expressive Individualism is an anthropology where the individual self is the fundamental unit of human reality. This self is not defined by its attachments or network of relations, but rather by its capacity to choose a future pathway that is revealed by the investigation of its own inner depths of sentiment. Key aspects of expressive individualism according to Snead include: (i) The self is not defined by objects of choice – whether property, a particular vocation, or even the creation of a family. (ii) The self is associated fundamentally with its will and not its body. (iii) Flourishing is achieved by turning inward to interrogate the self’s own deepest sentiments to discern the wholly unique and original truths about its purpose and destiny. (iv) The truth about the self is not determined externally, and sometimes must be pursued counter-culturally, over and above the mores of one’s community. (v) The self is bound only to those commitments freely assumed. Does this contemporary vision reflect the full lived reality of human embodiment, with all that it entails?

## Contemporary integration: nowadays’ concept of the human mind

The advent of advanced neuroimaging techniques, such as functional magnetic resonance imaging (fMRI) and electroencephalography (EEG), has allowed researchers to explore the neural correlates of mental processes. Neuroscientific data provide valuable insights into the biological underpinnings of cognition, emotion, and behavior.

Contemporary psychology and psychiatry have evolved from a neurocentric view of the human mind—born in ancient time with Alcmaeon of Croton and Hippocrates’ *De morbo sacro*—to incorporate a holistic understanding. Psychodynamic, cognitive-behavioral, and humanistic approaches offer diverse perspectives on mental health, considering both conscious and unconscious processes. The Diagnostic and Statistical Manual of Mental Disorders (DSM) reflects the ongoing effort to classify and understand psychiatric conditions.

According to psychiatrist and philosopher Thomas Fuchs, for example, there is a concrete and scientific-based alternative perspective to the prevailing naturalist view (or *neurocentric* view) that mental illnesses can be solely attributed to brain dysfunctions. Fuchs argues that mental illness cannot be reduced to mere brain dysfunction. His systemic and ecological account is based on three realities: (1) contextual understanding, *id est*, mental illness is inseparable from both the living organism and the patient’s life world or social environment. It cannot be isolated solely within the brain; (2) circular causality, *id est*, Fuchs proposes a shift from unilinear causation to circular causality. Mental disorders result from disruptions in both vertical circular causality (interplay between lower-level processes and higher faculties of the organism) and horizontal circular causality (social relationships and responsiveness to others); and finally, (3) brain mediation, *id est*, while brain processes play a role. Mental illnesses cannot be exclusively located within the brain. Reduction of mental illnesses to brain diseases is fundamentally challenging. Nowadays neuroscientific account teaches us the interconnectedness of mental illness with the whole person, their context, and circular causal processes beyond the brain. This perspective invites us to consider a more holistic understanding of mental health and a better 360-degree therapeutic interventions.^2^ The brain is interpreted as a subsystem of the person-system as a mediating organ:

Cognitive neuroscience has been driven by the idea that by reductionist analysis of mechanisms within a solitary brain one can best understand how the human mind is constituted and what its nature is. The brain thus came to appear as the creator of the mind and the experienced world. In contrast, the paper argues for an ecological view of mind and brain as both being embedded in the relation of the living organism and its environment. This approach is crucially dependent on a developmental perspective: the brain is conceived as a plastic system of open loops that are formed in the process of life and closed to full functional cycles in every interaction with the environment. Each time a new disposition of coherent neural activity is formed through repeated experience, structures of the mind are imprinted onto the brain. The brain becomes a mediating organ or a window to the mind, for it is structured by the mind itself.^3^

Acknowledging the relational dimension involves recognizing the impact of social, cultural, and interpersonal factors on mental well-being. From family systems theory to attachment theory, contemporary approaches highlight the importance of relationships in shaping psychological development and mental health.

Daniel J. Siegel, a prominent psychiatrist, clinical professor, and author, is known for his pioneering work in the field of interpersonal neurobiology. His holistic approach to understanding the human mind transcends traditional disciplinary boundaries, integrating neuroscience, psychology, and the social sciences. Siegel’s vision of the human mind is encapsulated in his concept of the mind itself, a term he uses to represent the embodied and relational nature of mental processes. Siegel emphasizes the idea of the mind as an emergent and embodied process that arises from the intricate interplay of the brain, the body, and the external environment. Siegel’s work is deeply rooted in the interdisciplinary field of interpersonal neurobiology.^4^ This approach integrates findings from neuroscience, psychology, and other disciplines to offer a comprehensive understanding of the mind. Siegel posits that the mind is not confined to the brain alone but is distributed throughout the body and is shaped by social interactions. Siegel emphasizes the embodied nature of the mind, acknowledging the interconnectedness of the brain and the body. He underscores the importance of integration, both within the brain itself (integration of different neural circuits) and between the individual and their social environment. Integration, according to Siegel, leads to a flexible and adaptive mind. The term *mindsight* refers to the capacity to perceive and understand one’s internal mental processes. It involves the ability to observe one’s thoughts and feelings without being overwhelmed by them. Mindsight, as conceptualized by Siegel, is crucial for mental well-being, fostering emotional regulation, empathy, and enhanced interpersonal relationships. Siegel often refers to the *triangle of well-being*, which highlights the interconnectedness of the mind, the brain, and relationships. This model underscores the idea that the mind is shaped by both internal and external factors, including biological processes, mental activities, and social interactions. Siegel embraces the concept of neuroplasticity, the brain’s ability to reorganize and adapt in response to experience. This idea reinforces the notion that the mind is not fixed but can be shaped and transformed through intentional mental practices, relationships, and experiences.^5^

This vision of the human mind is a holistic and integrative perspective that emphasizes the embodied, relational, and dynamic nature of mental processes. By weaving together insights from various disciplines, it provides a framework for understanding how the mind emerges from the intricate dance between the brain, the body, and the social world. This contemporary account of the body–mind problem sees the human person as a unity, neither a sole brain, nor a disembodied mind, but a third “thing”:

…[A human being] is said to be from soul and body as a third thing constituted from two things neither of which he is, for a [human] is not soul nor is he body.^6^

This medieval understanding indicates that one body plus one soul/mind equals a third, original material: the human being, which has a dual nature (not dualistic!). It is surprising that a contemporary holistic and integrative account of the human person sounds in the line of Thomas Aquinas’ consideration that it is not the body (or the brain) that “contains” the mind, but the mind the body.^7^

According to the well-known Italian philosopher of science Evandro Agazzi, the human mind is a complex subsystem of the whole person-system characterized – according to psychiatrist Daniel J. Siegel, Thomas Fuchs, and Georg Northoff – as an emergent, self-organizing, embodied, and relational (embeddedness) process that regulates the flow of energy and information of the organism. This definition suggests that the mind is not confined to or localized in the brain or even the physical body but is a systemic process that emerges from both the body and our relationships with others and the environment (epigenetic causality). It also implies that the mind is capable of self-organization and plays a crucial role in regulating how energy and information flow within us and between us and our environment. This perspective allows for a more comprehensive understanding of the mind, encompassing not just our thoughts, emotions, and memories but also our connections with others and the world around us.^8^

So, what it really means to be *human*, to be a human *person*? In contemporary neuroethical account – neuroethics is a systematic and informed reflection dealing with neuroscience and interpretations of the same neural sciences – the human person is not considered as a “separate and distinct from the manner in which he is or is not embedded in a web of social relations,” neither he/she is not identified with and defines by the exercise of their will – their capacity for choosing in accordance with their wants and desires”; the human person is not identical to the psychological conception of personhood that “decisively privileges cognition as the indispensable criterion for membership in this category of beings. In this way, it appears to be dualistic, distinguishing the mind from the body.” The mind and will alone do not define the whole of the human person, and the body is not merely “a contingent instrument for pursuing the projects that emerge from cognition and choice.”^9^

Public bioethics’ debates prevails a kind of individualism that is classified as *expressivism*, which elevates autonomy and liberty above the fundamental human good, *id est*, human life. As expressed by many philosophers, neuroscientists, physicians, and others, “human beings experience themselves and one another as living bodies, not disembodied wills”^10^:

the anthropology of the atomized, unencumbered, inward-directed self of expressive individualism falls short because it cannot render intelligible either the core human realities of embodiment or recognize the unchosen debts that accrue to all human beings throughout their life spans^11^ […]. Like Milton’s Satan and fallen angels, the expressive individual self “know[s] no time when [it was] not as now; Know none before [it], self-begot, self-rais’d/By [its] own quickening power.” A purely inward-looking and individualistic anthropology can give no intelligible or justified account of uncompensated, unconditional, and often self-sacrificial care of others. There is no warrant to give more than one could ever hope to receive. There is no imperative to give to those from whom nothing will ever be repaid in return.^12^

## Benefits for psychiatric treatment and psychological assessments

This holistic, integrative, and ecological vision of the human mind helps therapeutic approaches that combine physical and mental elements, such as exercise, yoga, meditation, biofeedback, and hypnosis. These solutions are based on the idea that the mind and the body are not separate, but rather interconnected and interdependent, and that they influence each other in various ways. These solutions aim to enhance the well-being and health of both the mind and the body by addressing the psychological, emotional, social, and biological factors that affect them; also, they offer a holistic and integrative way of treating and preventing mental disorders, such as depression, anxiety, stress, and addiction. These disorders are often associated with physical symptoms, such as pain, fatigue, insomnia, and inflammation, as well as cognitive and emotional impairments, such as memory loss, low self-esteem, and negative mood. This type of dual approach to the mind–body relation can help to alleviate these symptoms, by improving the physiological and psychological functioning of the mind and the body, and it can help to promote positive mental health, by enhancing the resilience, happiness, and quality of life of individuals. The mind–body solutions can foster a sense of self-awareness, self-regulation, and self-care, by teaching individuals how to cope with stress, manage their emotions, and cultivate positive habits and attitudes. This mind–body solution can also foster a sense of connection, belonging, and meaning, by facilitating social support, interpersonal relationships, and spiritual growth.^13^

This approach is supported by scientific evidence, which shows that they can have beneficial effects on the brain, the nervous system, the immune system, and the endocrine system. These effects can modulate the neurochemical and hormonal balance, the inflammatory and oxidative stress response, and the gene expression, which are involved in the development and maintenance of mental disorders. This mind–body solution can also influence the neural pathways, the brain regions, and the brain networks, which are involved in the regulation of cognition, emotion, and behavior, and it is not meant to replace the conventional treatments for mental disorders, such as medication and psychotherapy but rather to complement and enhance them. This mind–body solution can also offer a personalized and flexible way of addressing the specific needs and preferences of each individual, by taking into account their physical and mental condition, their personality and lifestyle, and their cultural and environmental context, and it can also empower individuals to take an active role in their own recovery and well-being, by providing them with skills and tools that they can use in their daily life.^4^

Thomas Fuchs offers a systemic and ecological account as an opposing view to the naturalist idea that mental illnesses can be reduced to dysfunctions of the brain. He regards mental illness as inseparable from the living organism and the patient’s life world or social environment. He introduces the notion of circular causality to replace the notion of monolinear causation in order to grasp mental disorders in their context. Fuchs identifies two types of disruptions that characterize mental illnesses: (i) vertical circular causality: this refers to the interplay between lower-level processes and higher faculties of the organism. This primarily affects a mentally ill person’s relation to themselves, which continually codetermines the course of the illness; (ii) horizontal circular causality: this refers to social relationships and the ability to respond adequately to the demands and expectations of others. Disruptions here lead to negative feedback loops in socio-functional cycles that influence the course of the illness from the very beginning. Both kinds of circular causal processes are tied to mediation by the brain but cannot exclusively be located within it. Therefore, Fuchs argues that reducing mental illnesses to diseases of the brain is in principle not possible.^14^ In his article *The Brain – A Mediating Organ*, Fuchs challenges the reductionist view of cognitive neuroscience that sees the brain as the creator of the mind and the experienced world and proposes an ecological view of the mind and brain, arguing that both are embedded in the relationship between the living organism and its environment. This perspective is dependent on a developmental view: the brain is seen as a plastic system of open loops formed in the process of life and closed to full functional cycles in every interaction with the environment. Each time a new disposition of coherent neural activity is formed through repeated experience, structures of the mind are imprinted onto the brain. Thus, the brain becomes a mediating organ or a window to the mind, as it is structured by the mind itself. Fuchs presents the brain not as the seat of the mind, but as an organ that mediates our conscious experience, structured by the mind through our interactions with the world.^15^

This integrated, holistic, and ecological solution is not only important for individuals but also for society, as they can contribute to the prevention and reduction of the burden and cost of mental disorders, which affect millions of people worldwide, and it can also foster a culture of health and wellness, by raising awareness and education about the importance and benefits of the mind–body connection, and by encouraging people to adopt healthy and positive behaviors and practices. Finally, it can also foster a culture of compassion and empathy, by promoting a holistic and humanistic view of mental health, and by reducing the stigma and discrimination that often surround mental disorders.

## Conclusion

The history of the concept of mind in the Western context has seen a dynamic interplay of philosophical, scientific, and psychological ideas. The integration of neuroscientific data, psychological and psychiatric orientations, and the relational dimension offers a comprehensive framework for understanding the human mind. By embracing a multidimensional perspective, this synthesis allows for a more nuanced and holistic-ecological approach to mental health, bridging the gap between the subjective and objective aspects of the human experience. As our understanding continues to evolve, this integrated concept of the human mind holds promise for advancing both theoretical knowledge and practical applications in the fields of psychology, psychiatry, and neuroscience.

In conclusion, the unidual body–mind solution sketched in this article is important in the context of mental health because it offers a comprehensive and integrative way of enhancing the well-being and health of both the mind and the body by addressing the multiple and complex factors that affect them. This contemporary, anticartesian and antineurosolipsism solution—that sees the human mind both embodied and embedded, is supported by scientific evidence, and it can complement and enhance the conventional treatments for mental disorders, and it can also benefit individuals and society, by promoting positive mental health, and by fostering a culture of health, wellness, compassion, and empathy.

## Footnotes

1 Plato. Alcibiades I, 130 C: ο άνθρωπος δεν είναι τίποτε άλλο παρά η ψυχή του, id est: human being is nothing but his soul (mind).

2 Plato. Alcibiades I, 129 A.

3 ‘This being premised, to find wherein personal identity consists, we must consider what PERSON stands for; - which, I think, is a thinking intelligent being, that has reason and reflection, and can consider itself as itself, the same thinking thing, in different times and places; which it does only by that consciousness which is inseparable from thinking, and, as it seems to me, essential to it: it being impossible for anyone to perceive without PERCEIVING that he does perceive. When we see, hear, smell, taste, feel, meditate, or will anything, we know that we do so. Thus, it is always as to our present sensations and perceptions: and by this everyone is to himself that which he calls SELF:—it not being considered, in this case, whether the same self be continued in the same or divers substances. For, since consciousness always accompanies thinking, and it is that which makes everyone to be what he calls self, and thereby distinguishes himself from all other thinking things, in this alone consists personal identity, i.e. the sameness of a rational being: and as far as this consciousness can be extended backwards to any past action or thought, so far reaches the identity of that person; it is the same self now it was then; and it is by the same self with this present one that now reflects on it, that that action was done.’

4 See the vast bibliography of neuroscientist, psychiatrist and philosopher Georg Northoff, Canada Research Chair in Mind, Brain Imaging and Neuroethics Georg Northoff.
